# Supplementary material for: A systems approach identifies Enhancer of Zeste Homolog 2 (EZH2) as a protective factor in epilepsy
Source: PLoS One. 2019 Dec 31;14(12):e0226733. doi: 10.1371/journal.pone.0226733 (PMC6938365; doi:10.1371/journal.pone.0226733)
Supplement: Attachment — Submitted filename: Response to reviewer comments.docx [file pone.0226733.s021.docx]

**Reviewer 1.**

***1. To identify the driver of transcriptional changes, the authors use transcriptomic profiles of dentate granular cells from three different rodent models with induced SE and confirm the changes in the expression of EZH2 in human samples from patients with TLE. Because these models do not cover all forms or causes of epilepsy, it is better to avoid discussing the observed changes in expression as a mechanism of epileptogenesis in general, unless the analysis is extended to other (including genetic) models of epilepsy***.

We agree and have adjusted our text. We now refrain from claiming that EZH2 drives epileptogenesis across all epilepsies.

***2. The variation in expression of EZH2 and other tested genes after KA induced seizures is performed for the first 30 days after SE (Figs 4 and 5). However, it would be interesting to also assess changes in the expression of EZH2 prior to and upon the occurrence of spontaneous seizures, especially since the data from human TLE samples indicate potential involvement in the chronic phase of epilepsy***.

This is a very interesting point. We have an ongoing project in the lab looking at how EZH2 changes as the animals move into the chronic phase. This is of particular importance as the reviewer points out because we see evidence of EZH2 function in human TLE. However, as the reviewer will appreciate, continuous monitoring of mice as they transition from a latent to chronic phase so that we may harvest brains at appropriate time points is a major undertaking and will be a publication in its own right. This issue is however, now discussed in the manuscript and we thank the reviewer for reminding us of its importance.

***3. The upregulation of EZH2 protein occurs transiently in the first 5 days post SE and returns to basal levels by day 10. Do the differences in the profile of repressed genes in 3 SE models between days 3 and 10 correspond to the changes in expression of five genes assessed using qPCR in the KA model? Are all analyzed genes in the group of shared repressed genes? How were the five genes selected for the analysis? – this should be described in more detail. Also the variability in their expression patterns should be addressed in the discussion***.

We now add more details about how these genes were chosen in the results section. In summary, we took all genes that were repressed at 1d and continued to be repressed at d10 post SE. We then took the 5 genes out of that set which had the highest EZH2 ChIP signals. The following text was added:

*“The genes were chosen on the basis that they have important functions in glutamatergic and GABAergic signaling, have been implicated in epilepsy disease pathology ^33,34^, Factor analysis tagged them as EZH2 targets and were repressed at 1d and out to 10d post SE across models in the EMC dataset.”*

**4. mRNA expression of EZH2 peaks at day 1 and day 10 post SE. While the first peak translates into increased protein expression at days 1-5, it is unclear whether the same happens after day 10 as the next analyzed time point is day 20. Adding a few time points between days 10 and 20 for the analysis of EZH2 protein expression and its impact on other genes would provide more complete profile of potential disease modifying effect and should be added to the paper**.

The complex pattern of EZH2 mRNA expression is now discussed in greater length. We do not know why EZH2 mRNA levels increase at 1 day, commensurate with protein levels, but then returns to baseline by 2 days only to transiently peak again at day 10. It is even more curious that this tracks with H3K27Me3 levels. This is elaborated upon in our updated discussion. The following text has been added to the discussion:

*“Results from the systemic KA and pilocarpine rodent models reinforce our computational predictions made from both rat and human transcriptomic data. We find that EZH2 protein levels manifest a prolonged increase after SE, which peaks at 2 days and remains increased out to 5 days. Interestingly, EZH2 mRNA was only transiently increased at 1d and 10d despite the largest increase in protein occurring in the intervening period. This suggests a complex regulation of EZH2 levels. An early increase in mRNA levels may contribute to increased EZH2 protein but much of the heightened EZH2 protein expression would have to be post-transcriptional. Whether this is due to decreased rates of degradation or increased rates of production awaits to be determined.*

*As the catalytic core of the PRC2 subunit, EZH2 catalyzes the trimethylation of H3K27^20^. We observe a peak of H3K27Me3 at 1d and 10d post SE but not in the intervening period when EZH2 levels are highest. This observation remains paradoxical but it is tempting to speculate that EZH2 may increase to super-stoichiometric levels compared to its’ obligate PRC2 subunits SUZ12 and EED whose levels do not change. This may render EZH2 unable to catalyze H3K27Me3 in the intervening period. This theory is the basis for ongoing work*.”

**5. Adding UNC1999 increases the frequency and severity of spontaneous seizures. Because the genes that are repressed by EZH2 are involved in synaptic transmission and neuronal excitability, knowing how the EZH2 inhibitor affects the activity of neurons and/or networks in this model may be a more relevant indicator of overall changes occurring in neurons upon inhibition of EZH2 than the provided profile of expression for a few selected genes**.

This is an excellent point and is the focus of ongoing work in the lab. However, the results from these studies are outside the scope of the current manuscript.

**Reviewer 2.**

**…the working hypothesis of this study is based on several outdated or flawed hypotheses:**

**1.) Existence of a latent period. This has not been observed in mice (Mazzuferi et al., 2012: doi: 10.1016/j.expneurol.2012.08.022). Furthermore, evidence shows that the frequency of spontaneous recurrent seizures is not a step-function of time (as implied by the hypothesis of a “latent period”). Seizure frequency is rater increasing as a sigmoid function of time after Kainate-induced SE (Williams et al., 2009: doi: 10.1523/JNEUROSCI.0980-08.2009). Interictal spikes during the latent period have been shown (Bortel et al., 2009: doi: 10.1016/j.nbd.2010.07.015).**

To argue that a latent period does not exist in mice is controversial and stretches the conclusions of the references the reviewer provides. Also, after claiming that no latent period exists, the reviewer writes *“…Interictal spikes during the latent period have been shown*.” If the reviewer believes that a latent period does not exist, how does he/she explain that interictal spikes can exist in a latent period?

The reviewer quotes a commentary from Michael Wong when he/she states:

“*as implied by [the hypothesis of] the “latent period”*” Epilepsy Currents, (2009 pp. 144–145)

I would point out that Wong wraps up the commentary by stating:

“*Thus, if epileptogenesis does continue beyond the latent period, then antiepileptogenic therapies may still have beneficial effects after presentation of the first seizure…”.*

Wong implicitly accepts the existence of a latent period but argues that epileptogenic processes continue after that period.

The Mazzarufi manuscript cited by the reviewer is limited to the pilocarpine mouse model; we used data from mouse pilocarpine, rat pilocarpine, mouse systemic kainate, rat systemic kainate, rat self sustaining status epilepticus and human data.

The Williams et al article cited by the reviewer argues that the latent period has complexities beyond a step function but does not come close to arguing for its absence. There is absolutely no question that a latent period exists in human epilepsy that can last for years to decades. In rodent models this can range from days to months. I leave out referencing this statement because the bibliography would run into the hundreds.

**2.) Existence of a protective activity following SE. This hypothesis is not supported by any substantial evidence. The ability to increase the seizure frequency following SE, shown in this and other studies, cannot be translated into the opposite direction.**

I refer the reviewer to figures 6A, 6B, 6C and 6D. The EZH2 inhibitor was administered for 3 days post SE. Video recording was performed 5 weeks after the last drug administration. The drug inhibits EZH2 and the animals have a greater seizure burden. Thus, animals that have uninhibited EZH2 have less seizure burden than animals with inhibited EZH2. Occam’s razor would suggest that given two competing hypotheses to explain a phenomenon, the simplest hypothesis is closer to the truth: The simplest explanation is that EZH2 is protective.

**Apart from this, there are several major issues:**

**I.) EZH2 is not found differentially downregulated or upregulated but is listed within the 9614 genes expressed in dentate granule cells. How is this to explain? How come then that EZH2 protein levels are found increased in mice after SE? Is it because of differences in time, SE induction model, mouse vs. rat model, experiment? These inconsistencies are not addressed.**

I would refer the reviewer to basic undergraduate texts in molecular biology. Many genes code for proteins whose levels do not track with mRNA levels. The fact that EZH2 is “*listed within the 9614 genes expressed in dentate granule cells*” means that the gene is expressed – a prerequisite for it’s levels to be increased. The disparity between EZH2 mRNA and protein levels is addressed in the discussion.

**II.) Usually, the first convulsive seizures are seen in rats after 5 days following SE (pilocarpine-induced, (Bortel et al., 2009: doi: 10.1016/j.nbd.2010.07.015). What this different for the rats of the Epilepsy Microarray Consortium Data? Were all granule cells collected before or after the onset of spontaneous seizures (specifically day 10)?**

The consortium data derives from pilocarpine, kainate and SSSE models using Sprague-Dawley rats. Each model presents with spontaneous seizures at different times after SE and even within a model there are lab to lab variations. The original collection of samples by the ECM was not performed in conjunction with seizure monitoring. The experimental conditions are described in Dingledine et al 2017.

**III.) Differential expressed genes at day 3 were not considered in any of the main analyses**.

GABRD, SC6A1, NTF3, SIDT1 and KCNK4 are all differentially expressed at day 3. I refer the reviewer to figures 1B, 1D, 1E, 4B, 4C, 4D, 5A, 5B, 5C for data on gene changes spanning or including day 3.

**IV.) What is the explanation for EZH2 as top3-TF for upregulated genes? This is briefly addressed in the manuscript but in a very unsatisfactory way**.

With as much respect as I can muster, I explain why I arrived at EZH2 being in the “*top3-TF for upregulated genes”* throughout the first half of the Methods section, throughout the Results section including figure 1, 2 and 3, and the bulk of the Discussion. This explanation is not “briefly addressed” but constitutes the bulk of the manuscript and is the entire drive for the paper.

**This result is actually, rather than having a biological explanation, pointing to a flawed statistical method to asses which transcription factors may be involved in the differential expression of a given set of genes**.

The statistical method pointed to EZH2 as a driver of gene changes post SE. This prediction was tested biochemically in 2 rodent models of acquired epilepsy. We performed western blots for EZH2 using post-SE hippocampi from a mouse kainate and rat pilocarpine model of acquired epilepsy and found a robust, prolonged >6 fold induction of the protein that lasted days after SE **EXACTLY AS OUR METHOD PREDICTED**. I can think of no other gene that is induced so robustly for so long in an epilepsy model. ***The method therefore, is not ‘flawed’. It made a perfect prediction***. If the reviewer can point to the flaw in the method - the entire mathematical basis of which is explicitly stated in the Methods section - I will be happy to address it. I will add that the reviewer’s assertion that all 11 authors who spent 6 years on this work were somehow fooled by flawed statistics is offensive.

**V.) No evidence in presented that upregulation of Ezh2 has any effects on the seizure frequency after SE**.

I refer the reviewer to figures 5B, 5C and 5D. Suppressing EZH2 activity increases seizure frequency after SE.

In summary, I thank the first reviewer for insightful and helpful comments that have improved the manuscript.
