# Supplementary material for: A systems approach identifies Enhancer of Zeste Homolog 2 (EZH2) as a protective factor in epilepsy
Source: PLoS One. 2019 Dec 31;14(12):e0226733. doi: 10.1371/journal.pone.0226733 (PMC6938365; doi:10.1371/journal.pone.0226733)
Supplement: Peer review history

# Author comment: Author response to previous submission

**DOI:** 10.1371/journal.pone.0226733.r001

**Transfer Alert**

This paper was transferred from another journal. As a result, its full editorial history (including decision letters, peer reviews and author responses) may not be present.

26 Nov 2019

Attachment Submitted filename: Response to reviewer comments.docx Click here for additional data file.

# Editor report: Decision Letter 0

**DOI:** 10.1371/journal.pone.0226733.r002

**Authors:** Gregory S. Barsh

6 Dec 2019

A Systems Approach Identifies Enhancer of Zeste Homolog 2 (EZH2) as a Protective Factor in Epilepsy.

PONE-D-19-32779

Dear Dr. Roopra,

Your manuscript has successfully been transferred to PLOS ONE and we are pleased to inform you that your manuscript has been judged scientifically suitable for publication. It will be formally accepted for publication once it complies with all outstanding technical requirements.

Within one week, you will receive an e-mail containing information on the amendments required prior to publication. When all required modifications have been addressed, you will receive a formal acceptance letter and your manuscript will proceed to our production department and be scheduled for publication.

Shortly after the formal acceptance letter is sent, an invoice for payment will follow. To ensure an efficient production and billing process, please log into Editorial Manager at https://www.editorialmanager.com/pone/, click the "Update My Information" link at the top of the page, and update your user information. If you have any billing related questions, please contact our Author Billing department directly at authorbilling@plos.org.

If your institution or institutions have a press office, please notify them about your upcoming paper to enable them to help maximize its impact. If they will be preparing press materials for this manuscript, you must inform our press team as soon as possible and no later than 48 hours after receiving the formal acceptance. Your manuscript will remain under strict press embargo until 2 pm Eastern Time on the date of publication. For more information, please contact onepress@plos.org.

With kind regards,

Gregory S. Barsh

Guest Editor

PLOS ONE

Reviewers' comments:

# Editor report: Acceptance letter

**DOI:** 10.1371/journal.pone.0226733.r003

**Authors:** Gregory S. Barsh

20 Dec 2019

PONE-D-19-32779 

A Systems Approach Identifies Enhancer of Zeste Homolog 2 (EZH2) as a Protective Factor in Epilepsy. 

Dear Dr. Roopra:

I am pleased to inform you that your manuscript has been deemed suitable for publication in PLOS ONE. Congratulations! Your manuscript is now with our production department. 

If your institution or institutions have a press office, please notify them about your upcoming paper at this point, to enable them to help maximize its impact. If they will be preparing press materials for this manuscript, please inform our press team within the next 48 hours. Your manuscript will remain under strict press embargo until 2 pm Eastern Time on the date of publication. For more information please contact onepress@plos.org.

For any other questions or concerns, please email plosone@plos.org. 

Thank you for submitting your work to PLOS ONE.

With kind regards,

PLOS ONE Editorial Office Staff

on behalf of

Dr. Gregory S. Barsh 

Guest Editor

PLOS ONE
